# Protocatechuic Acid Prevents Some of the Memory-Related Behavioural and Neurotransmitter Changes in a Pyrithiamine-Induced Thiamine Deficiency Model of Wernicke–Korsakoff Syndrome in Rats

**DOI:** 10.3390/nu15030625

**Published:** 2023-01-26

**Authors:** Kinga Krzysztoforska, Agnieszka Piechal, Ewa Wojnar, Kamilla Blecharz-Klin, Justyna Pyrzanowska, Ilona Joniec-Maciejak, Jan Krzysztoforski, Ewa Widy-Tyszkiewicz

**Affiliations:** 1 Department of Experimental and Clinical Pharmacology, Centre for Preclinical Research and Technology CePT, Medical University of Warsaw, Banacha 1b, 02-097 Warsaw, Poland; 2 Faculty of Chemical and Process Engineering, Warsaw University of Technology, Waryńskiego 1, 00-645 Warsaw, Poland

**Keywords:** protocatechuic acid, Wernicke–Korsakoff syndrome, memory, behaviour, neurotransmitters, glutamate

## Abstract

The purpose of this research was to investigate the effects of protocatechuic acid (PCA) at doses of 50 and 100 mg/kg on the development of unfavourable changes in cognitive processes in a pyrithiamine-induced thiamine deficiency (PTD) model of the Wernicke–Korsakoff syndrome (WKS) in rats. The effects of PCA were assessed at the behavioural and biochemical levels. Behavioural analysis was conducted using the Foot Fault test (FF), Bar test, Open Field test, Novel Object Recognition test (NOR), Hole–Board test and Morris Water Maze test (MWM). Biochemical analysis consisting of determination of concentration and turnover of neurotransmitters in selected structures of the rat CNS was carried out using high-performance liquid chromatography. PTD caused catalepsy (Bar test) and significantly impaired motor functions, leading to increased ladder crossing time and multiplied errors due to foot misplacement (FF). Rats with experimentally induced WKS showed impaired consolidation and recall of spatial reference memory in the MWM test, while episodic memory related to object recognition in the NOR was unimpaired. Compared to the control group, rats with WKS showed reduced serotonin levels in the prefrontal cortex and changes in dopamine and/or norepinephrine metabolites in the prefrontal cortex, medulla oblongata and spinal cord. PTD was also found to affect alanine, serine, glutamate, and threonine levels in certain areas of the rat brain. PCA alleviated PTD-induced cataleptic symptoms in rats, also improving their performance in the Foot Fault test. In the MWM, PCA at 50 and 100 mg/kg b.w. improved memory consolidation and the ability to retrieve acquired information in rats, thereby preventing unfavourable changes caused by PTD. PCA at both tested doses was also shown to have a beneficial effect on normalising PTD-disrupted alanine and glutamate concentrations in the medulla oblongata. These findings demonstrate that certain cognitive deficits in spatial memory and abnormalities in neurotransmitter levels persist in rats that have experienced an acute episode of PTD, despite restoration of thiamine supply and long-term recovery. PCA supplementation largely had a preventive effect on the development of these deficits, to some extent also normalising neurotransmitter concentrations in the brain.

## 1. Introduction

Cognitive impairment severely reduces the quality of life and makes it difficult to perform daily activities without assistance. The problem is affecting an increasing number of patients, which is primarily related to the lengthening human lifespan and the increased incidence of dementia and neurodegenerative diseases. However, cognitive impairment can also be triggered by other factors, such as malnutrition [1,2]. Therefore, any interventions that improve cognitive performance and prevent future memory deficits seem very important. Considering numerous current studies, anthocyanins and their metabolites have been shown to have beneficial effects on cognitive function [3,4].

Protocatechuic acid (PCA) is a widely distributed phenolic compound found in fruits, vegetables, plant-based beverages such as tea or wine, and cereals [3,5]. It is also formed in the intestines of animals as a major metabolite of anthocyanins and procyanidins and is largely responsible for their health-promoting effects, such as antioxidant, anti-inflammatory, neuroprotective, antibacterial, antidiabetic, antiatherosclerotic, antineoplastic and intensively studied procognitive effects [6,7,8]. PCA has been demonstrated to influence differentiation, proliferation and neuroprotection of cultured neuronal stem cells and increase cell viability in vitro [9]. PCA has been shown in vivo to have beneficial effects on memory and learning processes in D-galactose-induced memory impairment in rats [10], to alleviate cognitive deficits and attenuate inflammatory responses in AβPP/PS1 double transgenic mice with Alzheimer’s disease [8]. Moreover, PCA protects against neurotoxicity and oxidative stress induced by different toxins and heavy metals, e.g., cadmium in rats [11]. Furthermore, PCA inhibits the chronic intermittent hypoxia-induced apoptosis in hippocampus and prefrontal cortex in rats [6], reduces the global cerebral ischemia-induced hippocampal neuronal death [12], and favourably affects the concentration and/or metabolism of neurotransmitters (dopamine and serotonin) in the prefrontal cortex, hippocampus, and striatum of rats by counteracting changes induced by compounds that accelerate aging, induce oxidative stress, and enhance the production of advanced glycation end products (AGEs) [10].

Wernicke–Korsakoff syndrome (WKS) is a neuropsychiatric pathology associated with thiamine deficiency (TD), with two phases: acute—short-lived but severe Wernicke’s encephalopathy and chronic—amnestic, long-lasting, and debilitating Korsakoff’s psychosis, characterised by behavioural disturbances and profound memory impairment [13]. Currently, alcoholism accounts for most cases of WKS, but there are two common misconceptions about the prevalence of WKS in the population: that it is exclusive to alcoholics and that it is rare. As a matter of fact, patients after bariatric surgery, patients with eating disorders (e.g., anorexia nervosa) and malnourished (e.g., after long-term use of total parenteral nutrition without thiamine), with Crohn’s disease, AIDS, acute pancreatitis, kidney diseases, cancer, and even patients with diarrhoea or prolonged vomiting unrelated to alcohol consumption (e.g., hyperemesis gravidarum) are at risk of developing WKS [14]. The prevalence of WKS in the general population is 2%. It is about 12.5% in alcoholics, 10% in AIDS patients 10%, and 5.5% in patients after bone marrow transplantation, which proves that alcohol abuse is not a necessary condition for the development of WKS [15]. Brain structures that are particularly susceptible to damage in WKS include the thalamus, hypothalamus, mammillary bodies, brainstem, and cerebellum; neuronal loss in the prefrontal cortex has also been observed [16]. This causes people with WKS to have multiple cognitive deficits ranging from amnesia to dementia. The most striking distortions occur in recent memory, but sometimes long-remembered information is also affected [17]. Therefore, accompanying WKS cognitive dysfunction has important clinical consequences for many patients and should not be underestimated.

Thiamine plays the same role in both rodent and human brains, so many of the changes that accompany acute and chronic TD can be assessed using animal models. It has been proven that the pyrithiamine-induced thiamine deficiency (PTD) model of WKS results in the full clinical spectrum of WKS, including chronic cognitive impairment and Korsakoff’s syndrome. In the case of PTD, TD is generated by feeding thiamine-deficient chow in conjunction with daily i.p. injections of pyrithiamine (thiamine pyrophosphokinase inhibitor), which inhibits the metabolism and transport of thiamine through the blood–brain barrier [18,19]. The first symptom of TD in the PTD model is progressive weight loss, which typically becomes apparent on day 10 of the experiment. Over the next few days there is a slow and gradual decrease in motor activity, and usually at around 12–13 days it is followed by a loss of righting reflex (LRR), severe ataxia, and acute neurological disorders: generalised and myoclonic seizures, retropulsion, and opisthotonus. On days 13–16, the late acute stage of PTD can be observed, which corresponds to significant neuronal loss [20]. At this stage, parenteral administration of thiamine is essential to keep the rats alive. In addition to the loss of neurons in various areas of the brain, which remains even after thiamine supplementation and a well-balanced diet are restored, long-term neurochemical alterations in overall monoamines and amino acids were observed in animals following the PTD procedure [19].

The mechanism of cell damage following TD is complex. Thiamine plays an important role as a cofactor of enzymes involved in carbohydrate catabolism, amino acid accumulation, the pentose phosphate pathway, and consequently the production of neurotransmitters, glutathione, nucleic acids, coenzymes, steroids, and fatty acids. TD-dependent disturbances in carbohydrate metabolism may result in mitochondrial damage, increased levels of highly reactive molecules responsible for oxidative stress, and associated cell apoptosis [13]. Excessive production of reactive oxygen species, changes in superoxide dismutase levels, and excitotoxic amino-acid-mediated neuronal cell death have been observed in TD laboratory animals, suggesting that oxidative stress underlies the molecular mechanism of neurotoxicity in WKS [21]. Therefore, attempts to use antioxidant compounds (such as PCA) to prevent or treat WKS seem to be a logical approach.

This study for the first time analyses the effects of PCA on behavioural and biochemical markers of learning and memory in rats exposed to PTD, mimicking WKS, and assesses the neurological changes produced in a PCA-protected PTD-model of WKS.

## 2. Materials and Methods

### 2.1. Animals

Forty male Sprague Dawley rats (approximately 80 days old) weighing 238–342 g on arrival were obtained from the Medical University of Warsaw Animal Lab and acclimatised with free access to water and food. Animals were housed two per cage at controlled temperature (20–24 °C) and humidity (~60%). Their body weight was regularly monitored. The experiments were carried out in the light phase of the 1:1 light:dark cycle. All procedures were approved by the Ethical Committee for Animal Experiments at the Medical University of Warsaw in compliance with the EU Directive 2010/63/EU.

### 2.2. Drugs, Treatment, and Early Neurological Assessment

PCA (purity ≥ 97%), pyrithiamine hydrobromide (purity ~95%) and thiamine hydrochloride (purity ≥ 99%) were purchased from Sigma-Aldrich, Poznan, Poland. Labofeed standard laboratory diet was purchased from Factory of Fodder Morawski, Poland. Thiamine-deficient diet (thiamine contents < 2 mg/kg, cat. No. E15316-14) was manufactured by Ssniff Spezialdiäten GmbH, Soest, Germany.

**Figure 1:**
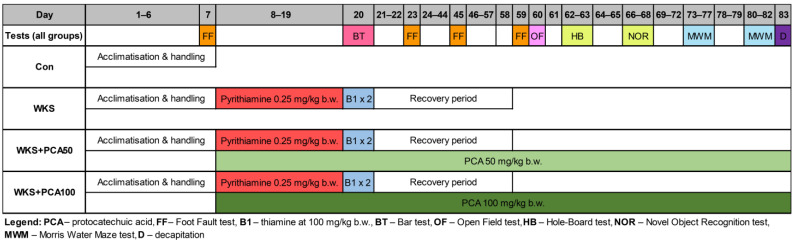
Experimental design, with timeline of treatments and behavioural testing.

Forty rats were randomly divided into four groups. The ‘Con’ group (*n* = 10) received a standard diet, tap water and no drugs, while the remaining 30 rats developed a PTD model of WKS due to administration of a thiamine-deficient diet (ad libitum) for 12 days with a single i.p. pyrithiamine injection (0.25 mg/kg b.w.) (see Figure 1). From the administration of the first dose of pyrithiamine, the animals underwent daily neurological assessment under the supervision of a clinical neurologist with many years of experience in experimental work with animals, to detect early the first symptoms of acute WKS, i.e., ataxia, loss of righting reflex (LRR), convulsions, opisthotonus. LRR was measured by failing to land on all four legs after being released upside down from 40 cm above a soft cushion, indicating sensory and motor impairment [22]. On day 13, when neurological signs became apparent, further pyrithiamine injections were discontinued and the rats were given two i.p. thiamine injections (100 mg/kg b.w.) 8 h apart. Along with thiamine injections, the thiamine-deficient diet was replaced with standard laboratory chow to avoid animal deaths and begin the 5-week recovery period necessary to regain strength and vitality for all rats before starting behavioural experiments that require fine motor skills. Of the 30 thiamine-deficient rats, 10 rats received no other treatment and served as model controls (‘WKS’ group), while the remaining 20 rats were co-administered with 50 or 100 mg/kg b.w. PCA in drinking water and were used to assess the impact of PCA on the development of PTD and further cognitive performance (groups ‘WKS + PCA50′ and ‘WKS + PCA100′, respectively). PCA supplementation in the respective groups was started with the first dose of pyrithiamine and continued until decapitation.

### 2.3. Assessment of Motor Function

#### Foot Fault Test

The Foot Fault test, also known as a ladder rung walking test, was conducted to assess the function of the animals’ limbs and walking coordination. The apparatus was placed 30 cm above the table surface and consisted of two transparent Plexiglas sidewalls (length 100 cm, height 20 cm) connected by removable metal rungs placed in holes drilled 1 cm above the bottom of the walls and forming an elevated horizontal ladder, as detailed in Metz and Whishaw (2002) [23]. The test was performed at four time points: before the first dose of pyrithiamine (Session 1), two days after the last dose of pyrithiamine (Session 2), and after 3-week and 5-week recovery periods, respectively (Sessions 3 and 4). One day prior to testing, the animals were trained to cross a ladder with rungs regularly spaced at 2-cm intervals. During the actual testing sessions, the position of the rungs changed from session to session and formed an irregular pattern (the distance between the rungs varied from 1 to 5 cm). During each session, the rats had two attempts to cross the ladder and reach the home cage located at the end of the apparatus, requiring them to carefully place their paws on the rungs between the holes. The cut-off time of the trial was 120 s. Each episode when the paw missed a rung or slipped and fell into the hole was counted as a foot fault (error). The stepping pattern was recorded taking into account the number of errors and the total time needed to complete the task (both averaged over two trials).

### 2.4. Assessment of Cataleptic Behaviour

#### Bar Test

The Bar test was performed to assess catalepsy, a behavioural state characterised by loss of control in the locomotor system and leading to a ‘frozen’ or stationary position. While a healthy rat placed in an uncomfortable position will quickly improve its posture, a cataleptic animal will remain in this imposed position for a longer period. The Bar test was performed 8 h after the first of the two doses of thiamine and consisted of placing the rat in an unusual position with the hindlimbs on the ground and forelimbs on a horizontal metal bar (diameter 1 cm) stretched between two supports at a height of 9 cm. Catalepsy was determined by the time both forelimbs of the animal remained on the bar. The trial was terminated when one forelimb was removed from the bar and touched the ground or after the 30 s cut-off time had elapsed. The time needed to correct the body posture was recorded and regarded as an index of the severity of catalepsy.

### 2.5. Cognitive Behaviour Evaluation

#### 2.5.1. Open Field Test (OF)

The OF originally developed by Hall (1934) [24] was performed after 40 days of recovery from PTD in a square apparatus with surrounding walls (30 cm high, 100 × 100 cm) made of Plexiglas and brightly lit. The computer tracking system used for data recording (EthoVision XT 10, Noldus) invisibly divided the floor into two zones: central (78 × 78 cm) and peripheral. Each rat was placed individually in the arena for a 3-min session. Each time before a rat was placed in the apparatus, the floor was washed with a 10% ethanol solution to get rid of scent marks left by other animals.

#### 2.5.2. Hole–Board Test (HB)

The HB originally developed by Boissier et al. (1962) [25] and used to study animals’ emotionality, stress response, anxiety, but also locomotor activity and speed of movements after exposure to a new environment was performed after 42 days of recovery from PTD. The apparatus was similar to the OF apparatus but contained 16 evenly spaced holes in the floor (four rows of four holes ø 4 cm). A 10% ethanol solution was used to eliminate the scent marks left by animals. The test was tracked with an EthoVision XT 10 (Noldus).

#### 2.5.3. Novel Object Recognition Test (NOR)

The NOR, first described by Ennaceur and Delacour (1988) [26], was performed after 46 days of recovery from PTD in the OF apparatus. On the first day, habituation took place in an empty arena (30 cm high, 100 × 100 cm). On the second day (familiarisation), two identical objects A (green, vertical block) or B (blue, horizontal block) were placed in opposite corners of the arena and each rat was allowed to explore them for 3 min. To eliminate the risk of preference of one type of object, half of the rats in each group explored the arena with two objects of type A and the other half with two objects of type B. On the third day (making choices), the rats explored an apparatus containing one familiar and one novel object. Exploration was considered the moment the animal sniffed or touched the object. After each session, the blocks were cleaned with 10% ethanol to remove any traces of scent. The test was recorded (EthoVision XT 10, Noldus). The memory discrimination index was calculated as follows: (N − F)/(N + F), where N is the time spent exploring a new object, and F a known object. The higher the memory discrimination index, the greater the memory retention for the familiar object.

#### 2.5.4. Morris Water Maze Test (MWM)

After 53 days of recovery from PTD, rats were subjected to the MWM, first described by Morris (1984) [27], to assess hippocampus-dependent spatial learning and memory. The method was based on the procedure of Widy-Tyszkiewicz et al. (2002) [28] in a circular pool (ø 150 cm) filled up to 30 cm with water (23 °C) and surrounded by visual cues for spatial orientation. The pool was divided into quadrants: north-east (NE), north-west (NW), south-east (SE), and south-west (SW). During the test, the rats learned the location of an escape platform located in the SE quadrant and submerged 1 cm below the water surface. On days 1–4 (acquisition), the animals were given one training session of four trials daily. For each trial, rats were placed in the water facing the pool wall at one of three equally spaced starting points, excluding the quadrant containing the platform, to avoid initial proximity to the platform. The trial was terminated when the rat entered the platform (transfer latency). Rats that failed to find the escape platform within 60 s of swimming were given a 60 s score and were placed on the platform before starting the next trial. On day 5 (probe trial), the platform was removed, and the rats were allowed to swim in the pool for 60 s. On day 8 (after a two-day break) a recall was conducted (the platform was placed back in the SE quadrant) followed by training on day 9 with the platform placed in the NW quadrant. A second memory test was performed on day 10 to reveal platform preference (old vs. new position). As a control protocol, a test with a clearly marked platform was performed. Data were recorded using a video camera connected to an image-analysing system (EthoVision XT 10, Noldus). Depending on the day of the test, the swimming speed, total distance travelled, transfer latency, crossings over the platform position, cumulative time spent in different parts of the pool, and the number of faecal pellets were assessed in the MWM. Prior to the actual test, the rats were habituated to a different pool to reduce the stress effect.

### 2.6. Sample Preparation

The day after the MWM, the rats were sacrificed by decapitation, the brains were removed and immediately transferred to an ice-cold plate. The hypothalamus, hippocampus, prefrontal cortex, medulla oblongata and spinal cord were immediately collected, weighed, frozen on dry ice and stored at −80 °C according to the method described by Glowinski and Iversen (1966) [29] until used for further biochemical analysis. Prior to analysis, samples of the dissected brain structures were homogenised in 1000 μL of a mixture containing ice-cold 0.1 M perchloric acid and 0.05 mM ascorbic acid using an ultrasonic cell disruptor, and then centrifuged at 13,000× *g* for 15 min at 4 °C to precipitate proteins. After filtration using membrane syringe filters (0.2 μm pore size, Whatman-Cytiva, Marlborough, MA, USA), the supernatant was collected.

### 2.7. Biochemical Evaluation

#### 2.7.1. Quantification of Monoamines

For the determination of dopamine (DA), its metabolites 3,4-dihydroxyphenylacetic acid (DOPAC), homovanillic acid (HVA), serotonin (5-HT), its metabolite 5-hydroxyindoleacetic acid (5-HIAA), and noradrenaline (NA) and its metabolite 3-methoxy-4-hydroxyphenylglycol (MHPG), 20 μL of the supernatant was injected into high-performance liquid chromatography with electrochemical detection (HPLC-ED) according to the method of Widy-Tyszkiewicz et al. (2002) [28]. The apparatus consisted of a delivery pump (Mini-Star K-500), an autosampler injector (L-7250, Merck-Hitachi), a column and an amperometric detector (L-3500 A, LaChrom) with a glassy carbon electrode. Separation was performed using a C-18 reverse phase analytical column with a particle size of 5 μm (Nucleosil, Macherey-Nagel). The mobile phase flow rate was 0.8 mL/min. The potential of the detector electrode was +0.8 V in relation to the Ag/AgCl electrode. The mobile phase consisted of a citrate-phosphate buffer (32 mM disodium phosphate, 34 mM citric acid, 1 mM octanesulfonic acid and 54 μM ethylenediaminetetraacetic acid (EDTA), Sigma-Aldrich) in deionised ultrapure water (18 MΩ·cm) containing 16% methanol (Merck). Monoamine/metabolite concentrations were quantified by comparison with standard reference solutions (Sigma-Aldrich) run before and after each set of tissue samples.

#### 2.7.2. Quantification of Amino Acids

Samples after analysis of monoamines were used to measure amino acid concentrations. Concentration of taurine (TAU), histidine (HIS), serine (SER), aspartic acid (ASP), alanine (ALA), glutamic acid (GLU), γ-aminobutyric acid (GABA) and threonine (THR) in the hypothalamus, hippocampus, prefrontal cortex, medulla oblongata and spinal cord of rats were analysed using HPLC-ED and pre-column derivatisation with ophthaldialdehyde based on the method of Rowley et al. (1995) [30]. The apparatus consisted of a delivery pump (Primade 1110, Merck-Hitachi), an autosampler (Primade 1210, Merck-Hitachi), a column (Luna C18(2), 250 mm length × 4 mm internal diameter; particle size: 5 μm; Phenomenex), and a glassy carbon electrode detector (EC Recipe 3000, Merck). The mobile phase consisted of 45 mM disodium phosphate dihydrate, 0.15 mM EDTA (both from Sigma-Aldrich) and 24% methanol (Merck). The flow rate was 0.8 mL/min. To obtain the derivatising agents, 22 mg of o-phthaldialdehyde was diluted in a mixture of 0.5 mL of 1 M sodium sulphite, 0.5 L of methanol, 0.9 mL of 0.1 M sodium tetraborate buffer (pH 10.4 adjusted with 5 M sodium hydroxide). Before being applied to the column, 20 μL of derivatising agent was reacted for 15 min with the amino acid standard and supernatant samples at room temperature. Samples were quantified by comparison with standard reference solutions (Sigma-Aldrich). Amino acids concentrations were calculated using Primade (Merck).

### 2.8. Statistical Analysis

Statistical analysis was performed using STATISTICA 13.1 (StatSoft) and RStudio software. Results are presented as mean ± standard error of the mean (SEM). Differences between groups were assessed using analysis of variance (ANOVA) preceded by an analysis of equality of variance (Levene’s test). In case of violation of the assumption of equal variance, the Welch’s correction for ANOVA was applied (results are marked with a ‘W’ in superscript). Comparisons between groups were performed using the post-hoc Newman–Keuls test (NK) for equal variances and the Games–Howell test (GH) for unequal variances. Repeated Measures ANOVA was used to analyse repeated observations. Differences were considered significant at *p* < 0.05. The number of animals in each of the four groups studied throughout the experiment was *n* = 10.

## 3. Results

### 3.1. Neurological Assessment and Body Weight

**Figure 2:**
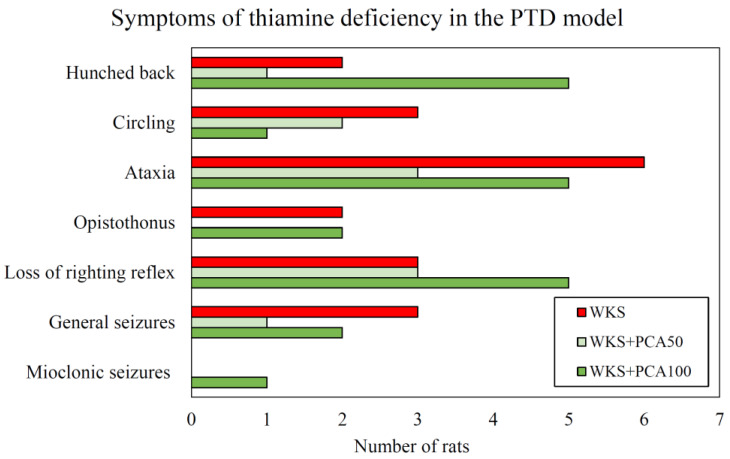
The frequency of neurological symptoms recorded in animals during the period of pyrithiamine administration. The control group (no pyrithiamine and, hence, no PTD symptoms) is not visualised.

The frequency of individual neurological symptoms recorded in animals during the administration of pyrithiamine (for WKS, WKS + PCA50, and WKS + PCA100 group) is shown in Figure 2.

**Figure 3:**
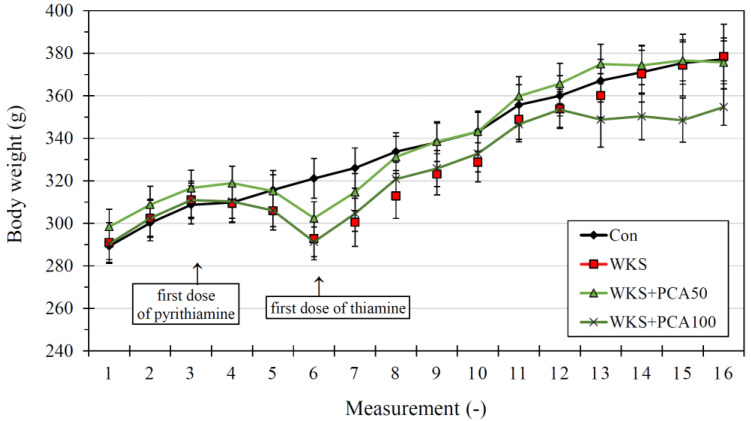
Changes in body weight (means ± SEM) in rats from the control group (Con), the PTD-induced WKS group (WKS), and the PTD-induced WKS groups treated with PCA at doses of 50 mg/kg (WKS + PCA50) or 100 mg/kg (WKS + PCA100) during the experiment. Between the administration of the first dose of pyrithiamine and the first dose of thiamine, there was a period of thiamine deficiency lasting 12 days (not applicable to control group).

The body weight of the rats on arrival was: 289.30 ± 8.11 g (Con), 291.00 ± 9.31 g (WKS), 298.40 ± 8.22 g (WKS + PCA50), and 290.40 ± 7.43 g (WKS + PCA100), and at the end of the experiment it was: 377.1 ± 10.06 g (Con), 378.40 ± 15.22 g (WKS), 375.70 ± 10.11 g (WKS + PCA50), and 354.70 ± 8.60 g (WKS + PCA100). There were no significant differences between the groups in both baseline (F_(3, 36)_ = 0.25, *p* > 0.05) and final (F_(3, 36)_ = 0.99, *p* > 0.05) body weight. Changes in body weight of rats during the experiment are shown in Figure 3.

### 3.2. Foot Fault Test

#### 3.2.1. Accuracy of Foot Placement

**Figure 4:**
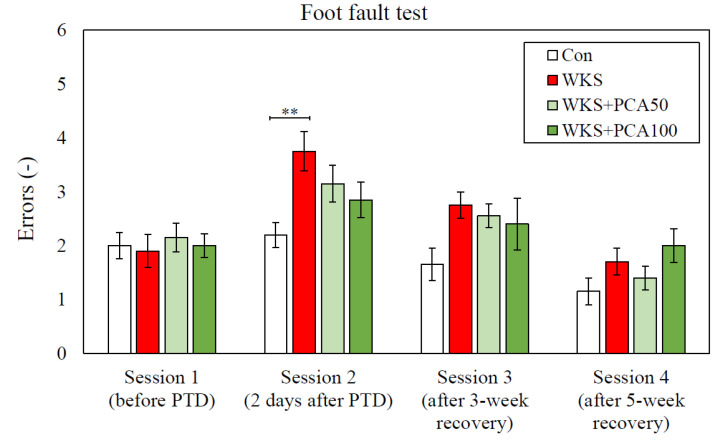
Number of errors (mean ± SEM) per session in Foot Fault test, ** *p* < 0.01 vs. Con (ANOVA, post-hoc NK).

In Session 1, performed before the administration of the first dose of pyrithiamine, there were no significant differences between the groups in the number of errors per session, which was an indicator of correct foot positioning. In Session 2, performed two days after the last dose of pyrithiamine, ANOVA showed significant differences between the groups in the number of errors (F_(3, 36)_ = 4.07, *p* = 0.014). The number of errors in WKS rats was the highest and significantly increased compared to the control group (Con: 2.20 ± 0.23 vs. WKS: 3.75 ± 0.36, *p* < 0.01, post-hoc NK). However, after both 3 and 5 weeks of recovery (Sessions 3 and 4), there were no differences between the groups in the number of foot faults (Figure 4).

#### 3.2.2. Time Measurements

**Figure 5:**
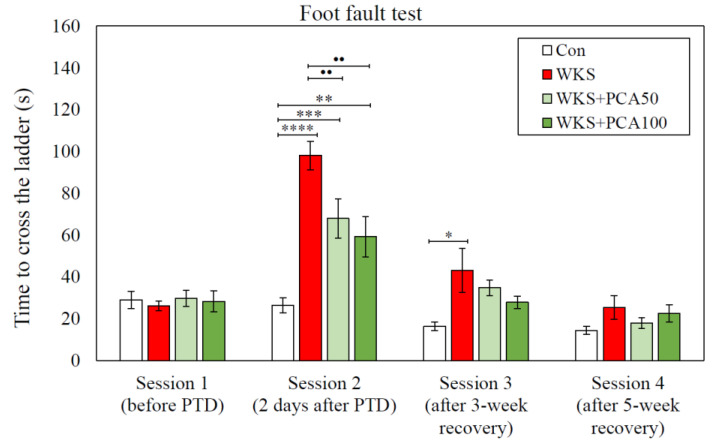
Time taken to cross the ladder (mean ± SEM) per session in Foot Fault test, * *p* < 0.05 vs. Con, ** *p* < 0.01 vs. Con, *** *p* < 0.005 vs. Con, **** *p* < 0.001 vs. Con, •• *p* < 0.01 vs. WKS.

The total time taken to cross the ladder in Session 1 was similar in all groups of animals. In Session 2, rats from each group significantly differed in the total time needed to complete the task (F_(3, 36)_ = 14.39, *p* = 0.000003). WKS rats took more than three times longer than control rats to cross the ladder (Con: 26.35 ± 3.64 s vs. WKS: 98.10 ± 6.85 s, *p* < 0.001, post-hoc NK). Moreover, in Session 2, PCA at both tested doses had a positive effect on the time needed to cross the ladder. As a result, the time to cross the ladder for both WKS + PCA50 and WKS + PCA100 rats was shorter than for WKS rats (WKS: 98.10 ± 6.85 s vs. WKS + PCA50: 67.9 ± 9.43 s and WKS: 98.10 ± 6.85 s vs. WKS + PCA100: 59.25 ± 9.64 s, both *p* < 0.01, post-hoc NK). In WKS rats, an extended time to cross the ladder, as compared to control group, was also found in Session 3, despite a 3-week recovery period (Con: 16.3 ± 1.96 s vs. WKS: 43.05 ± 10.48 s, *p* < 0.05, post-hoc GH). However, in Session 4, after a full 5-week recovery, the time to cross the ladder was similar in all groups (Figure 5).

#### 3.2.3. Bar Test

**Figure 6:**
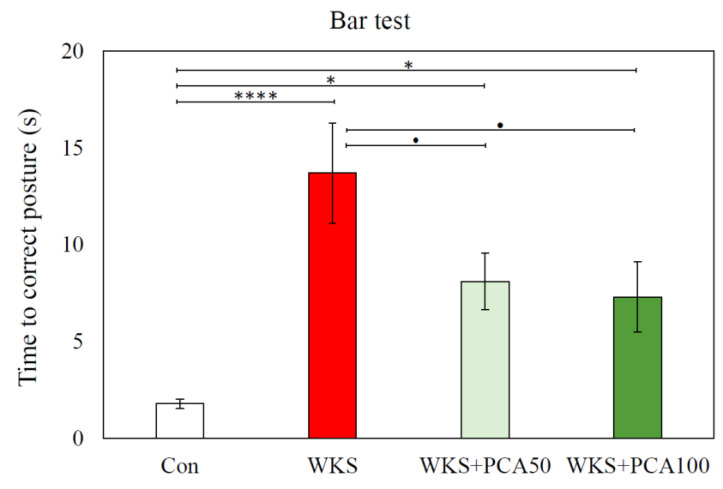
Time (mean ± SEM) needed to correct the imposed body posture in the Bar test, * *p* < 0.05 vs. Con, **** *p* < 0.001 vs. Con, • *p* < 0.05 vs. WKS (ANOVA, post-hoc GH).

In the Bar test, ANOVA revealed significant differences between the groups in the time needed to correct the externally imposed body posture (F_(3, 15.54)_ = 14.23^W^, *p* = 0.001). In WKS rats, the postural correction time was the longest and more than seven times longer than in control rats (Con: 1.80 ± 0.25 s vs. WKS: 13.70 ± 2.60 s, *p* < 0.001, post-hoc GH). In addition, PCA at both tested doses was shown to reduce the severity of catalepsy (WKS: 13.70 ± 2.60 s vs. WKS + PCA50: 8.10 ± 1.46 s and WKS: 13.70 ± 2.60 s vs. WKS + PCA100: 7.30 ± 1.81 s, all *p* < 0.05, post-hoc GH) (Figure 6).

### 3.3. Open Field Test

In the OF, there were no significant differences between the groups in terms of total time spent in the central zone (F_(3, 36)_ = 1.24, *p* > 0.05), the number of entries to the central zone (F_(3, 36)_ = 0.44, *p* > 0.05), and total time spent in the peripheral zone (F_(3, 36)_ = 1.24, *p* > 0.05). The groups also did not differ in the number of grooming episodes understood as licking or scratching the body while motionless (F_(3, 36)_ = 2.36, *p* > 0.05), rearing episodes (F_(3, 36)_ = 1.78, *p* > 0.05), number of faecal boli produced (F_(3, 36)_ = 2.05, *p* > 0.05), number of micturitions (F_(3, 36)_ = 0.53, *p* > 0.05), and time spent in motion (F_(3, 36)_ = 2.24, *p* > 0.05). ANOVA showed significant differences between the groups in total distance travelled (F_(3, 36)_ = 3.07, *p* > 0.040) and average walking speed (F_(3, 36)_ = 3.24, *p* > 0.033), but further post-hoc analysis showed that in both cases there were differences only between the control group of healthy rats and the WKS + PCA100 group. Detailed results are presented in Table 1.

### 3.4. Hole–Board Test

In the HB, neither the first- nor the second-day ANOVA with post-hoc tests showed significant differences between the control group and the WKS model group. There was also no statistically significant effect of PCA on the behaviour of animals or the results obtained by them in the PTD model of WKS. Detailed results for the parameters analysed in the test are presented in Table 2.

### 3.5. Novel Object Recognition Test

#### 3.5.1. Familiarisation

In the NOR familiarisation phase, ANOVA showed no significant differences between the groups neither in the total exploration time of the first (F_(3, 36)_ = 1.44, *p* > 0.05) and second object (F_(3, 36)_ = 2.02, *p* > 0.05), time of first contact with first (F_(3, 36)_ = 1.90, *p* > 0.05) and second object (F_(3, 36)_ = 1.51, *p* > 0.05), nor the number of contacts with first (F_(3, 36)_ = 0.36, *p* > 0.05) and second object (F_(3, 36)_ = 1.30, *p* > 0.05). The compared groups did not differ significantly in the total exploration time of both objects (F_(3, 36)_ = 1.42, *p* > 0.05) and the total number of contacts with the objects (F_(3, 36)_ = 0.71, *p* > 0.05).

#### 3.5.2. Recognition

During the NOR recognition phase, when rats had to choose between the familiar and the novel object, ANOVA did not reveal significant differences between the groups in the total exploration time of the novel object (F_(3, 36)_ = 1.74, *p* > 0.05), time of first contact with the novel object (F_(3, 36)_ = 0.48, *p* > 0.05), and the number of contacts with novel object (F_(3, 36)_ = 2.57, *p* > 0.05). There were also no significant differences between the groups in the same parameters describing the exploration of a familiar object. The total exploration time of both objects (F_(3, 36)_ = 0.69, *p* > 0.05) and the total number of contacts with both objects (F_(3, 36)_ = 1.61, *p* > 0.05) in the recognition phase were similar for all study groups. At first glance, the memory discrimination index describing the recognition memory in the NOR was significantly lower in the WKS rats than in the other groups (more than ten times lower than in the control group), but ANOVA showed no significant differences between the groups in the value of this index (F_(3, 36)_ = 1.52, *p* > 0.05).

### 3.6. Morris Water Maze Test

#### 3.6.1. Acquisition

**Figure 7:**
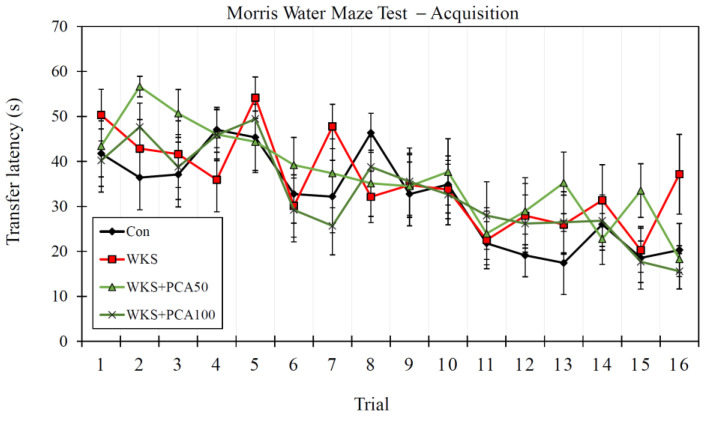
Learning progress in MWM acquisition phase as expressed by transfer latencies during successive trials of a 4-day training (mean ± SEM; four trials per day).

ANOVA of the results obtained by the rats during the acquisition phase of MWM, which included sixteen swimming trials to locate the underwater platform for each rat in the first four days of the experiment (four swimming attempts a day), showed no significant differences between the groups in swimming speed (F_(3, 156)_ = 1.16, *p* > 0.05), total distance travelled to find the platform (F_(3, 156)_ = 0.63, *p* > 0.05), and transfer latency (F_(3, 156)_ = 1.40, *p* > 0.05). There were also no differences between the groups in learning progress, defined as a gradual reduction in latency times as the rats made successive swimming attempts, which was measured by Repeated Measures ANOVA (F_(3, 36)_ = 0.61, *p* > 0.05) (Figure 7).

#### 3.6.2. Recall of Memory

**Figure 8:**
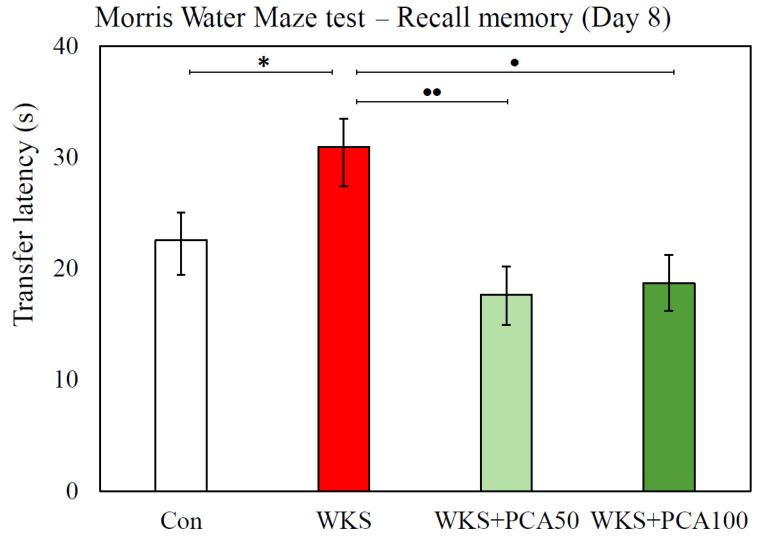
Transfer latency (mean ± SEM) in memory recall task in the MWM test, * *p* < 0.05 compared to Con, • *p* < 0.05 compared to WKS, •• *p* < 0.01 compared to WKS (ANOVA, post-hoc GH).

The recall test was conducted after a two-day break from swimming. After a break, attempts were resumed to find the underwater platform placed in the same position as before in the SE quadrant of the pool. ANOVA showed significant differences between the groups in the transfer latency needed to locate the platform (F_(3, 86.09)_ = 3.46^W^, *p* = 0.020). Rats in the WKS group took almost 1.5 times longer to recall the platform location than rats in the control group (WKS: 30.92 ± 3.50 s vs. Con: 22.55 ± 3.10 s, *p* < 0.05, post-hoc GH). Animals given PCA at both doses performed significantly better than WKS rats and found the platform in a time similar to that of control rats (WKS: 30.92 ± 3.50 s vs. WKS + PCA50: 17.66 ± 2.75 s, *p* < 0.01, and WKS: 30.92 ± 3.50 s vs. WKS + PCA100: 18.69 ± 2.51 s, *p* < 0.05, post-hoc GH) (Figure 8). However, the compared groups of animals did not significantly differ in swimming speed (F_(3, 156)_ = 2.27, *p* > 0.05) and the total distance travelled to the platform (F_(3, 156)_ = 0.96, *p* > 0.05).

#### 3.6.3. Working Memory in Reversal Training

During reversal training, when the underwater platform was moved to the NW quadrant and the rats were tasked with remembering its new location, there were no statistically significant differences between the test groups in swimming speed (F_(3, 156)_ = 0.62, *p* > 0.05), total distance travelled to the platform (F_(3, 156)_ = 1.60, *p* > 0.05), and transfer latency (F_(3, 156)_ = 1.36, *p* > 0.05). In addition, by dividing the latency value obtained by each animal during the second swim trial in the training session with the platform placed in a new location by the latency value obtained during the first swim trial (TL2/TL1), an index describing working memory was calculated. There were no significant differences between the study groups in the value of this index (F_(3, 36)_ = 2.61, *p* > 0.05).

#### 3.6.4. Consolidation

**Figure 9:**
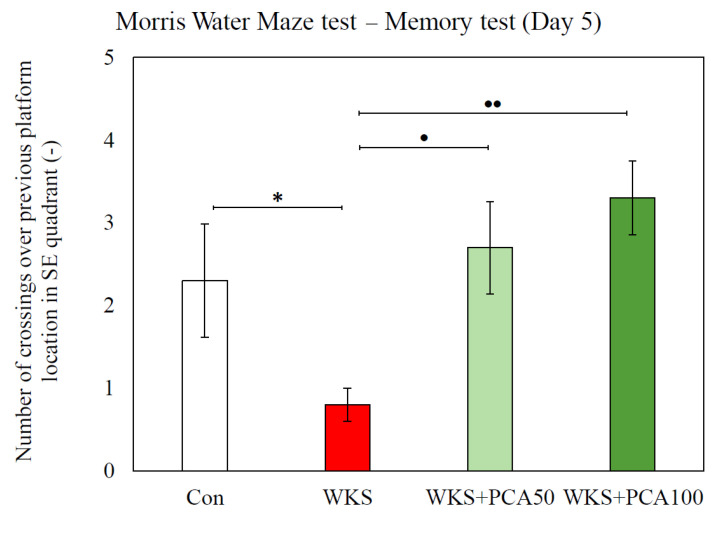
Number of crossings (mean ± SEM) over the previous platform location during the first memory test in MWM, post-hoc GH. * *p* < 0.05 compared to Con, • *p* < 0.05 compared to WKS, •• *p* < 0.01 compared to WKS.

**Figure 10:**
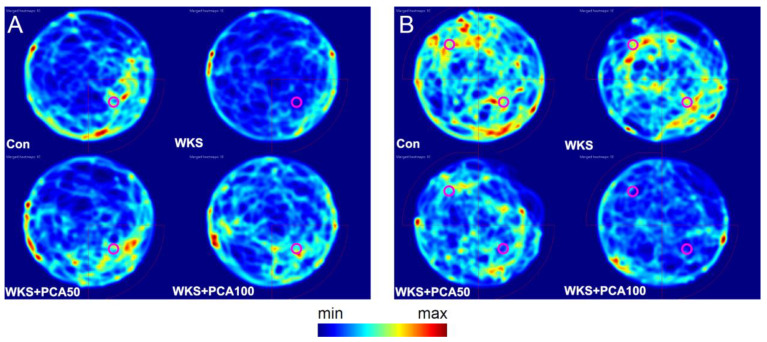
Heat maps showing the spatial distribution of time spent by each group of animals at each pool location: (**A**) in the first memory test and (**B**) in the second memory test in MWM. The circle slices represent the SE and NW target quadrants along with platform location during the training phases (red circles in the lower right or upper left corner of each map).

The first memory test conducted on the fifth day of the MWM test assessed spatial reference memory consolidation in rats. The measure of memory consolidation was the ability to successfully identify the location in the pool where the underwater platform was placed during the four-day acquisition training. For this purpose, the number of crossings over the previous platform location was assessed. ANOVA showed significant differences in the number of crossings between the groups (F_(3, 17.77)_ = 10.57^W^, *p* = 0.0003). The number of crossings in WKS rats was almost three times lower than in control rats, indicating a significant impairment of spatial memory consolidation caused by the thiamine-deficiency episode (WKS: 0.80 ± 0.20 vs. Con: 2.30 ± 0.68, *p* < 0.05, post-hoc GH). In contrast, the number of crossings in the WKS + PCA50 and WKS + PCA100 groups was significantly higher than for the WKS group (WKS: 0.80 ± 0.20 vs. WKS + PCA50: 2.70 ± 0.56, *p* < 0.05 and WKS: 0.80 ± 0.20 vs. WKS + PCA100: 3.30 ± 0.45, *p* < 0.01, post-hoc GH) and did not differ significantly from the number of crossings in the control group, indicating a beneficial effect of PCA at both tested doses on spatial memory consolidation (Figure 9). However, the analysis of the cumulative time spent in the target SE and other quadrants of the pool showed no significant differences between the groups. The distribution of time spent at each pool location in first memory test is shown in the heat map (Figure 10A).

A second memory test conducted on the tenth day to assess the memory of the new vs. old platform location showed no statistically significant differences between the groups, neither in the number of crossings over areas in SE and NW quadrants where the platform was previously located nor in the cumulative time spent in each pool quadrant. The distribution of time spent at each pool location in the second memory test is shown in the heat map (Figure 10B). Moreover, none of the memory tests performed showed statistically significant differences between the control group and the WKS group in terms of swimming speed, total distance travelled, total time spent in the central vs. peripheral part of the pool, or the number of defecations.

#### 3.6.5. Control Protocol with Visible Platform

In the control protocol of searching for a clearly marked platform protruding above the water surface, there were no significant differences between the groups in terms of swimming speed (F_(3, 156)_ = 1.67, *p* > 0.05), transfer latency (F_(3, 156)_ = 2.87, *p* > 0.05), or total distance travelled (F_(3, 156)_ = 1.30, *p* > 0.05). Thus, no deviations between the compared groups of rats that could negatively affect the performance of spatial tasks by the animals were detected.

### 3.7. Brain Monoamines

ANOVA with subsequent post-hoc tests showed that significant differences between the control group and the WKS group in the concentration of monoamines and their metabolites concerned the concentration of 5-HT in the prefrontal cortex, the concentration of MHPG in the prefrontal cortex and medulla oblongata, and the concentration of HVA in the spinal cord and medulla oblongata. In all cases, the concentration of monoamine or its metabolite in the WKS group was significantly lower than in the control group. Significant differences were also found between the WKS group and the control group in the HVA/DA ratio in the medulla oblongata; this ratio was significantly lower in the WKS group. In none of the above cases was PCA found to have a significant effect on preventing these PTD-induced changes, and in the case of MHPG levels in the prefrontal cortex, PCA at a dose of 50 mg/kg further enhanced the effect of PTD. Post-hoc analysis also showed that PCA at 50 mg/kg significantly increased 5-HT (5-HIAA/5-HT) turnover in the hypothalamus and prefrontal cortex, although there was no difference between the control group and the WKS group. Detailed data are presented in Table 3.

### 3.8. Brain Amino Acids

**Figure 11:**
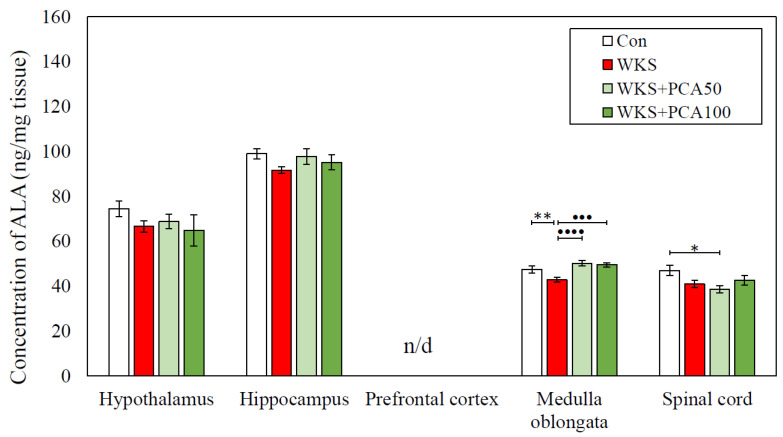
Alanine concentration (mean ± SEM) in selected CNS structures in rats from the control group (Con), the PTD-induced WKS group (WKS), and the PTD-induced WKS groups administered PCA at doses of 50 mg/kg (WKS + PCA50) or 100 mg/kg (WKS + PCA100); * *p* < 0.05 vs. Con, ** *p* < 0.01 vs. Con, ••• *p* < 0.005 vs. WKS, •••• *p* < 0.001 vs. WKS, n/d—no data (concentration below detection threshold) (ANOVA, post-hoc NK).

**Figure 12:**
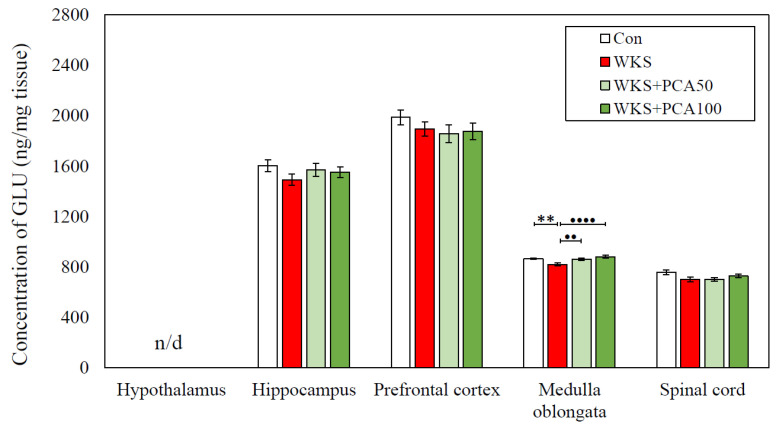
Glutamate concentration (mean ± SEM) in selected CNS structures of rats from the control group (Con), the PTD-induced WKS group (WKS), and the PTD-induced WKS groups administered PCA at a dose of 50 mg/kg (WKS + PCA50) or 100 mg/kg (WKS + PCA100); ** *p* < 0.01 vs. Con, •• *p* < 0.01 vs. WKS, •••• *p* < 0.001 vs. WKS (ANOVA, post-hoc NK).

ANOVA with subsequent post-hoc analysis revealed several statistically significant differences in amino acid concentrations between the WKS group and the control group. In the case of the WKS group, these differences each time consisted of a decrease in amino acid concentration in the examined CNS structure in relation to the control group and concerned the concentrations of serine in the spinal cord, the concentrations of alanine and glutamic acid in the medulla oblongata, and the concentration of threonine in the medulla oblongata, spinal cord, and hippocampus. Importantly, in the case of alanine and glutamic acid concentrations in the medulla oblongata, PCA at both doses tested showed a beneficial effect in preventing PTD-induced changes. Detailed data are presented in Table 4 and in Figure 11 and Figure 12.

## 4. Discussion

Cognitive dysfunction accompanying age-related diseases has recently been discussed in the context of aging in highly developed societies. It may occur as a result of vascular lesions, neurodegenerative processes, infections of the nervous system, intracranial proliferative processes, exposure to certain drugs and toxins, as well as hormonal and vitamin deficiencies, including TD. In alcoholics, TD is associated with the pathophysiology of WKS mainly because excessive alcohol consumption leads to insufficient food intake, but alcohol also reduces the absorption of thiamine from the gastrointestinal tract and impairs its storage and utilisation by cells. However, it is now known that many clinical situations unrelated to alcohol consumption lead to TD and the development of cognitive impairment. The adverse changes in the CNS associated with TD are complex and include increased concentrations of highly reactive molecules (free radicals) responsible for oxidative stress, decreased production of neurotransmitters, and disturbances in carbohydrate metabolism as a result of abnormalities in enzymatic pathways controlled by thiamine. The pathophysiology of WKS also includes excitotoxicity and inflammatory processes leading to neuronal cell death. Given the nature of WKS and its causes, it seems reasonable to evaluate the potential benefits of polyphenolic compounds in TD-induced cognitive impairment. The action profile of polyphenols, known for their antioxidant and neuroprotective activity, especially taking into account the reports of their effects on cognition and neurotransmission, indicates the possibility of obtaining therapeutic benefit. Other authors studying the potential mechanisms underlying WKS also recognise the need for neuroprotective therapeutic approaches [31]. The aim of this study was to shed light on the above issues and identify memory-related neurobehavioral and neurochemical effects of the potent polyphenolic compound PCA in PTD-induced WKS in rats, thus complementing recent promising reports on PCA. The presented results provide interesting conclusions about the properties of PCA and its role in counteracting the negative effects of TD on cognitive processes.

In the experiment, rats were given pyrithiamine injections and thiamine-free chow, which resulted in the development of neurological symptoms consistent with WKS. The symptoms gradually subsided after the injections were discontinued, the deficient chow was replaced with normal food, and thiamine was administered. In the Bar test performed after the end of the PTD episode after the first dose of thiamine, all rats treated with pyrithiamine exhibited cataleptic behaviour, the severity of which was significantly reduced by the co-administration of PCA at both 50 and 100 mg/kg. Catalepsy is known to be triggered by a variety of drugs that interfere with dopaminergic transmission, dopamine synthesis and storage, glutamatergic transmission, that are μ-opioid receptor agonists, muscarinic cholinergic agonists, or that interact with adenosine receptors [32]. The assessment of the motor functions of the forelimbs and hind limbs using the Foot Fault test showed that shortly after the end of the PTD episode (in Session 2), the WKS rats had difficulties in moving along the ladder, expressed in both a longer time to move along the ladder and a significantly higher number of error steps compared to the control group, even though both groups scored similarly in the same test before the PTD episode (in Session 1). Moreover, even after a three-week recovery period following an episode of PTD (in Session 3), the animals in the WKS group took a longer time to cross the ladder than the control rats. Administration of PCA at both 50 and 100 mg/kg b.w. significantly reduced the time to cross the ladder in Session 2. There is evidence that Foot Fault test scores are very sensitive to CNS damage. The test protocol allows the assessment of fine motor skills by analysing the animal’s limb positioning, stepping and coordination between limbs. The test can be useful to demonstrate both loss and recovery of sensorimotor functions related to brain and spinal cord injury, as well as the effectiveness of treatment [23,33]. In this study, the results showed that pyrithiamine treatment leading to TD is associated with sensorimotor impairment and poorer performance in assessing smooth locomotor skills. The manifestation of sensorimotor impairment in WKS rats three weeks after the PTD period demonstrated the advantage of the Foot Fault test, which is its sensitivity to detect even slight changes in sensorimotor performance, even at late time points, whereas in other tests the animals often learn to compensate for deficits in repeated test sessions. However, according to the test results, five weeks of a normal diet enabled rats given pyrithiamine to fully regain their limb coordination. Sensorimotor deficits were no longer detectable in the Foot Fault test, which allows us to conclude that the differences between individual groups of animals detected in subsequent behavioural memory tests resulted from cognitive, not motor deficits. The changes in behaviour and motor function in rats observed in the Bar test and Foot Fault test may be related to the impairment of dopaminergic and glutamatergic transmission in the thiamine-deficient animals observed in this study and discussed below.

An open-field study of rats after a full five-week recovery period showed no differences between control rats and thiamine-deficient rats in spontaneous exploratory behaviour and locomotor activity. Similarly, in the Hole–Board test, temporal deprivation of thiamine did not change the behaviour of the rats and did not inhibit their exploratory activity. The number of head-dippings and rearing episodes, as well as the time spent on moving did not significantly differ between tested groups of animals.

Interestingly, in animals in which WKS was experimentally induced with the thiamine-deficient diet and pyrithiamine, there were no differences in object recognition in the NOR, used to assess episodic memory, compared to control rats. Deficits in recognition memory leading to impaired ability to discriminate between a new and a familiar object in a rat model of WKS obtained by forced consumption of 20% alcohol were previously described by Moya et al. (2022) [31], but the same authors did not observe these deficits in animals in which WKS was induced by thiamine-deficient diet and pyrithiamine injections, which is consistent with the results obtained in the present study. This may indicate that alcohol-induced episodic memory impairment is not related to TD and is rather due to alcohol disrupting the function of the medial prefrontal cortex and other cortical structures that are recruited during memory recognition tasks and are susceptible to alcohol-induced chronic toxicity.

Many studies have shown that in thiamine-deprived animals, certain neuroanatomical, neurochemical, and behavioural changes persist from weeks to months after the recovery period, and some of these changes appear to be permanent [19]. In this study, the long-term effects of TD were observed in rats in the MWM test performed after a 7-week recovery from a TD episode. In a memory test conducted after a 4-day acquisition training, rats from the WKS group showed poorer recall of the position of the underwater platform, which was manifested by fewer crossings over the learned position of the platform, indicating an impaired spatial reference memory consolidation. In turn, the number of crossings over the previous position of the platform was increased in rats supplemented with PCA at both 50 and 100 mg/kg b.w., which indicates that PCA improves memory consolidation, which is consistent with Krzysztoforska et al. (2019) [10], who showed a positive effect of PCA at a dose of 100 mg/kg on spatial reference memory consolidation in rats in a D-galactose-induced model of memory impairment. In the paper mentioned above, and in the present study of PCA-treated rats, an increase in the number of crossings over the remembered platform location in the memory test was not accompanied by an increase in the cumulative time spent in the target SE quadrant of the pool. However, the increase in the number of crossings alone may indicate a more accurate recall of the platform’s location. Further significant PTD-induced changes were observed on day eight of the MWM test. They demonstrated impaired memory recall in rats with WKS. These rats showed almost 1.5 times longer latencies in finding the platform in the recall test, suggesting impaired information retention after learning. Also in this case, a positive effect of PCA at doses of 50 and 100 mg/kg was observed on preventing the adverse effect induced by PTD. In rats that experienced an episode of TD, but were given PCA, the results obtained in the recall test were similar to those in the healthy control group. An analogous effect of PCA consisting of the improvement of spatial memory retention and the reversal of unfavourable changes in memory performance was also described by the already cited Krzysztoforska et al. (2019) [10].

In patients with WKS, the areas of the brain where magnetic resonance imaging (MRI) typically detects changes in signal intensity are the medial thalamus, mammillary bodies, periaqueductal area, and tectal plate, but neuropathology studies have shown that changes also occur in unusual locations such as the cerebellum, medulla oblongata, spinal cord, and cerebral cortex [34,35]. In this study, the behavioural changes caused by PTD were accompanied by disturbances in serotonergic, dopaminergic, and noradrenergic neurotransmission in the brain, which were observed in the prefrontal cortex, medulla oblongata and spinal cord. Poorer results obtained by rats from the WKS group in the MWM test assessing the spatial reference memory were reflected in reduced 5-HT concentrations in the prefrontal cortex, which were not observed in the hippocampus or any other of the assessed brain structures. 5-HT imbalances in the prefrontal cortex, especially low levels of 5-HT, correlate with impaired memory consolidation and poor retention of information after learning [36]. This is consistent with the results of this study and is further supported by the results of a placebo-controlled double-blind crossover study by Harrison et al. (2004) [37], in which declarative memory consolidation was impaired due to reduced 5-HT synthesis in the brain, and the results of Haider et al. (2005) [38], where reduced levels of 5-HT in the brain due to lead toxicity correlated with deficits in spatial working memory. In turn, studies of selective serotonin reuptake inhibitors have shown that enhanced serotonergic transmission improves the performance of many declarative memory tasks in both animals and humans, prevents the development of pharmacologically induced amnesia, and improves memory consolidation and retrieval in mice [39,40]. In this study, a clearly beneficial effect of PCA on behavioural parameters indicating improved memory in the MWM test was observed. However, no effect of PCA on the normalisation of altered 5-HT levels was found, in contrast to Krzysztoforska et al. (2019) [10]. The only demonstrated effect of PCA on serotonergic transmission was that PCA at 50 mg/kg increased the turnover of PTD-unaltered serotonin turnover (5-HIAA/5-HT) compared to the WKS group, but this was significantly different from the effect of the higher dose. The study also showed long-term changes in neurotransmission induced by PTD, which did not subside after a recovery period and concerned the concentration of the DA HVA metabolite. In the WKS group, HVA concentration in the medulla oblongata and spinal cord was significantly decreased as a result of thiamine deficiency. In the medulla oblongata, this also translated into reduced DA turnover, expressed by the HVA/DA ratio, which may suggest that pyrithiamine-exposed rats had less intensified DA metabolism. The concentration of the NA metabolite MHPG was also altered in the WKS group, although the level of MHPG was decreased in the medulla oblongata and increased in the prefrontal cortex compared to the control group. In the prefrontal cortex, this effect was further enhanced by PCA at a dose of 50 mg/kg b.w.

This study also provides information on the effect of PCA on amino acid levels in pyrithiamine-induced WKS. In the CNS, amino acids serve as neurotransmitters and have neuropharmacological properties, and an imbalance of excitatory and inhibitory amino acids in the brain under abnormal conditions is implicated in the development of neurological disorders. In this experiment, PTD caused significant changes in the concentrations of serine, glutamate, alanine, and threonine. In all cases, the concentration of amino acids in the WKS group was decreased. The most striking changes in amino acids levels were observed in the medulla oblongata and spinal cord. In the medulla oblongata, PCA increased alanine and glutamate concentrations, preventing the pyrithiamine-induced changes. As a result, rats given PCA in addition to pyrithiamine at both tested doses showed alanine and glutamate concentrations that are not different from normal controls. PCA had no effect on serine and threonine concentrations. Surprisingly, amino acid levels in the prefrontal cortex and hypothalamus of pyrithiamine-treated rats were intact and similar to controls.

The results reported in this study partially coincide with those described by other authors, e.g., Langlais and Mair (1990) [22], who found significant reductions in glutamate in various brain regions, including mammillary bodies, hypothalamus, and hippocampus in rats with PTD-induced WKS. Decreased glutamate levels, particularly those persisting despite recovery from PTD, suggest that glutamate may be particularly involved in PTD-induced neuroanatomical changes, which may persist for weeks to months after thiamine deprivation or even be permanent. Proper glutamatergic transmission is generally believed to be profoundly affected in all major neuropsychiatric diseases. Glutamate homeostasis is controlled by the processes of astroglial glutamate release and uptake, and its disruption underlies neurotransmission imbalance, failure of information processing by neuronal networks, and is associated with the pathogenesis of many neuropsychiatric diseases. Impaired glutamate transmission and glutamate excitotoxicity also represent one of the major pathogenic factors in the development of WKS [41]. This is further supported by the findings that pre-treatment with N-methyl-D-aspartate (NMDA) receptor antagonist MK-801 has a protective effect on pyrithiamine-induced amino acid changes in the rat brain and blocks glutamate rise in the extracellular fluid, thereby significantly reducing pathologic damage in neuronal cells of WKS rats [42]. The normalising effect of PCA at 50 and 100 mg/kg on glutamate and alanine concentrations found in this study may therefore suggest its influence on glutamate-signalling astrocytic glutamate transporters 1 (EAAT1, GLAST) and 2 (EAAT2, GLT-1), which enable tight control of glutamate levels and whose loss or blockage is associated with oxidative stress and structural brain damage in WKS [21]. Recently, upregulation of glutamate transporters by polyphenol-rich *Hibiscus sabdariffa* extract was found to protect HT-22 cells from glutamate-induced neurodegeneration [43]. There are also reports that alanine is involved in the transport and storage of glutamate in astrocytes and glutamatergic neurons [44]. In this study, PCA had no effect on histidine levels, although postmortem examinations in healthy rats given PCA p.o. for 48 days showed a significant effect of PCA on the reduction of histidine concentrations in the hippocampus and prefrontal cortex of animals [45].

Animal studies have shown that PCA may be helpful in preventing or treating conditions related to neurocognitive dysfunction. These studies mainly focus on the antioxidant and anti-inflammatory effects of PCA, as well as its effects on synaptic plasticity, neural cell apoptosis and levels of neurotrophic factors in the brain. The present work adds to existing knowledge by providing information on the effects of PCA on memory performance, exploratory behaviour and motor function, and brain neurotransmitters in PTD rats. The presented results allow us to conclude that PCA may be helpful in preventing or mitigating the adverse processes associated with TD, which is increasingly being diagnosed in various clinical situations that at first glance seem unrelated to it.

## 5. Conclusions

The results presented herein indicate that pyrithiamine-induced thiamine deficiency in rats leads to severe neurological symptoms that mimic WKS and are very pronounced soon after an episode of thiamine-deficiency, but gradually regress when thiamine is introduced into the diet and pyrithiamine supply is discontinued. Finally, in animals deprived of thiamine, after a period of convalescence, no changes in the motor functions of the front and hind paws are observed, pre-existing neurological symptoms disappear, and in tests assessing exploratory, locomotor activity or episodic memory, no deviations from the control group are observed. However, animals treated with pyrithiamine also experience long-term or even permanent changes in cognitive functions that relate to the consolidation and recall of spatial reference memory and can be captured by the MWM test. These cognitive deficits observed in pyrithiamine-treated rats may be related to altered brain monoamine transmission, especially decreased cortical 5-HT levels, as well as alterations in DA turnover, as it is believed that appropriate 5-HT and DA signalling in the brain plays a key role in learning and memory processes. Abnormal levels of the amino acids serine, alanine, glutamate, and threonine, which modulate learning and memory in the brain, and which are found in WKS rats, may further contribute to the poor memory performance.

The obtained results also provide interesting conclusions about the protective effect of PCA on the development of cognitive impairment in PTD-induced WKS in rats. PCA at doses of 50 and 100 mg/kg b.w. administered to rats during the TD episode and during the recovery period effectively inhibited the development of long-term disorders of memory consolidation and recall, which were found in MWM test in animals not receiving PCA supplementation. In the presented study, PCA had a slight effect on the concentration of monoamines in the brain of the tested animals, while in the medulla oblongata it had a normalising effect on the concentrations of alanine and glutamate which were changed in animals with WKS that were not administered PCA. The obtained results indicating the protective effect of PCA in preventing the development of adverse changes in cognitive functions correspond to the results obtained by other researchers.

The mechanism by which PCA beneficially affects memory processes in the PTD model of WKS cannot be clearly explained, due to the complex nature of neurotransmission processes; however, the effect on glutamate concentration appears to be relevant. The antioxidant, antiplatelet or anti-atherosclerotic effects of PCA cannot be ruled out, which certainly also contribute to the beneficial effect of the compound on brain function. Other effects, such as epigenetic modulation of gene expression, are also possible as dietary phenolic-targeted epigenetics for disease prevention, and intervention is being intensively studied. Given that thiamine deficiency accompanies numerous and often unexpected clinical conditions, and the promising results regarding the beneficial effects of PCA on cognitive functions, these issues deserve further research.

## Figures and Tables

**Table 1:** Effect of long-term oral administration of PCA at doses of 50 mg/kg or 100 mg/kg on the behaviour of rats in the Open Field test in PTD model of WKS.

Group	Con	WKS	WKS + PCA50	WKS + PCA100
Total distance travelled (m)	**18.39 ± 0.84**	15.79 ± 1.61	14.71 ± 0.99	13.25 ± 1.37 *
Average walking speed (m/s)	**0.10 ± 0.00**	0.09 ± 0.01	0.08 ± 0.01	0.08 ± 0.01 *
Total time spent in the central zone (s)	42.27 ± 5.18	44.99 ± 4.53	52.20 ± 5.19	62.29 ± 13.58
Number of entries to the central zone (-)	10.10 ± 0.77	9.70 ± 1.47	10.90 ± 2.47	8.20 ± 1.69
Total time spent in the peripheral zone (s)	137.73 ± 5.18	135.01 ± 4.53	127.80 ± 5.19	117.71 ± 13.58
Number of rearing episodes (-)	7.50 ± 0.67	5.80 ± 1.10	5.10 ± 0.95	4.90 ± 0.75
Number of faecal boli produced (-)	0.50 ± 0.34	1.50 ± 0.54	0.20 ± 0.20	0.60 ± 0.40
Time spent in motion (%)	78.39 ± 1.29	73.04 ± 2.96	72.88 ± 2.30	66.01 ± 5.50
Number of grooming episodes (-)	0.50 ± 0.22	0.00 ± 0.00	0.10 ± 0.10	0.30 ± 0.15
Number of micturitions (-)	0.30 ± 0.21	0.10 ± 0.10	0.10 ± 0.10	0.10 ± 0.10

* *p* < 0.05 vs. Con.

**Table 2:** Effect of long-term oral administration of PCA at doses of 50 mg/kg or 100 mg/kg on the behaviour of rats in the Hole–Board test in PTD-model of WKS.

	**DAY 1**			
**Group**	**Con**	**WKS**	**WKS + PCA50**	**WKS + PCA100**
Number of head-dippings (-)	9.60 ± 1.22	9.90 ± 1.73	10.60 ± 0.95	9.10 ± 1.18
Number of rearing episodes (-)	3.50 ± 0.60	3.10 ± 0.87	3.60 ± 1.01	2.00 ± 0.49
Number of micturitions (-)	0.40 ± 0.27	0.30 ± 0.21	0.20 ± 0.13	0.00 ± 0.00
Number of faecal boli produced (-)	0.40 ± 0.40	0.00 ± 0.00	0.00 ± 0.00	0.00 ± 0.00
Number of grooming episodes (-)	0.10 ± 0.10	0.20 ± 0.13	0.10 ± 0.10	0.40 ± 0.16
Total distance travelled (m)	16.86 ± 0.95	15.92 ± 1.55	16.48 ± 1.21	26.20 ± 4.19
Average walking speed (m/s)	0.10 ± 0.01	0.09 ± 0.01	0.09 ± 0.01	0.15 ± 0.02
Time spent in motion (%)	72.73 ± 1.12	70.00 ± 3.02	67.95 ± 3.11	64.29 ± 2.33
Rotation clockwise (-)	2.50 ± 0.43	2.50 ± 0.43	2.30 ± 0.56	4.10 ± 1.27
	**DAY 2**			
**Group**	**Con**	**WKS**	**WKS + PCA50**	**WKS + PCA100**
Number of head-dippings (-)	7.50 ± 1.88	11.80 ± 2.15	10.70 ± 1.39	10.40 ± 1.17
Number of rearing episodes (-)	**3.30 ± 0.68**	1.40 ± 0.54	1.70 ± 0.63	1.10 ± 0.31 *
Number of micturitions (-)	0.40 ± 0.22	0.10 ± 0.10	0.00 ± 0.00	0.10 ± 0.10
Number of faecal boli produced (-)	0.30 ± 0.30	0.40 ± 0.40	0.50 ± 0.40	0.00 ± 0.00
Number of grooming episodes (-)	0.60 ± 0.27	0.30 ± 0.15	0.40 ± 0.16	0.30 ± 0.15
Total distance travelled (m)	14.25 ± 1.68	14.10 ± 1.20	12.56 ± 1.32	14.19 ± 0.64
Average walking speed (m/s)	0.08 ± 0.01	0.08 ± 0.01	0.07 ± 0.01	0.08 ± 0.00
Time spent in motion (%)	61.81 ± 3.36	64.33 ± 2.79	57.92 ± 4.48	64.53 ± 1.86
Rotation clockwise (-)	2.10 ± 0.43	3.10 ± 0.80	1.80 ± 0.36	1.60 ± 0.37

* *p* < 0.05 vs. Con.

**Table 3:** Effect of long-term oral administration of PCA at doses of 50 mg/kg and 100 mg/kg on the level of monoamines, their metabolites (mean ± SEM) (ng/g of tissue) and the turnover (-) in selected brain regions in the PTD model of WKS in rats.

Monoamine	Group	Hypothalamus	Hippocampus	Prefrontal Cortex	Medulla Oblongata	Spinal Cord
5-HT	Con	**815.47 ± 66.05**	461.47 ± 48.13	**477.33 ± 28.64**	687.08 ± 14.99	455.76 ± 9.54
	WKS	708 ± 47.94	434.49 ± 32.5	414.87 ± 19.11 *	724.00 ± 14.27	481.16 ± 61.39
	WKS + PCA50	578.1 ± 36.33 *	574.03 ± 66.07	355.49 ± 15.19 ****	713.67 ± 10.89	425.77 ± 12.26
	WKS + PCA100	644.03 ± 74.56	475.7 ± 56.04	383.09 ± 15.78 **	693.17 ± 15.12	445.77 ± 7.9
5-HIAA	Con	859.69 ± 76.28	430.51 ± 20.11	367.38 ± 21.04	437.71 ± 10.23	257.94 ± 9.6
	WKS	750.95 ± 28.68	360.24 ± 18.17	403.68 ± 24.47	429.55 ± 14.47	270.29 ± 23.81
	WKS + PCA50	897.56 ± 30.52	412.45 ± 24.73	436.32 ± 20.46	433.52 ± 13.31	246.9 ± 9.35
	WKS + PCA100	753.3 ± 87.59	373.23 ± 30.41	364.44 ± 18.24	408.70 ± 15.89	253.95 ± 9.77
5-HIAA/	Con	**1.15 ± 0.17**	1.03 ± 0.11	**0.8 ± 0.07**	0.64 ± 0.02	0.57 ± 0.02
5-HT	WKS	**1.12 ± 0.1**	0.85 ± 0.04	1.01 ± 0.1	0.59 ± 0.01	0.58 ± 0.02
	WKS + PCA50	1.6 ± 0.09 *•	0.78 ± 0.07	1.24 ± 0.05 •****	0.61 ± 0.01	0.58 ± 0.02
	WKS + PCA100	**1.19 ± 0.07 ^**	0.84 ± 0.07	**0.96 ± 0.05 ^**	0.59 ± 0.03	0.57 ± 0.02
DA	Con	174.28 ± 35.21	36.45 ± 9.04	20.18 ± 2.55	54.60 ± 1.33	46.47 ± 2.24
	WKS	195.77 ± 40.41	41.47 ± 9.52	18.09 ± 3.71	57.55 ± 2.55	41.32 ± 1.93
	WKS + PCA50	147.48 ± 24.83	72.65 ± 29.57	16.75 ± 2.63	54.89 ± 1.11	41.58 ± 1.38
	WKS + PCA100	121.12 ± 37.38	79.25 ± 48.13	12.74 ± 1.73	54.78 ± 0.99	41.68 ± 1.26
DOPAC	Con	66.22 ± 9.66	21.15 ± 5.64	27.56 ± 3.94	21.79 ± 1.48	8.03 ± 0.67
	WKS	107.95 ± 15.34	17.71 ± 3.7	32.25 ± 3.35	20.8 ± 0.74	8.10 ± 0.84
	WKS + PCA50	119.39 ± 15.58	63.62 ± 36.09	36.92 ± 2.85	20.75 ± 1.41	7.14 ± 0.32
	WKS + PCA100	70.88 ± 15.18	21.13 ± 8.27	25.37 ± 2.32	19.76 ± 0.62	6.08 ± 0.4
DOPAC/	Con	0.53 ± 0.12	0.58 ± 0.04	**1.42 ± 0.18**	0.40 ± 0.02	0.18 ± 0.02
DA	WKS	0.74 ± 0.12	0.44 ± 0.04	2.31 ± 0.32	0.36 ± 0.01	0.20 ± 0.02
	WKS + PCA50	0.91 ± 0.08	0.52 ± 0.11	2.62 ± 0.37 *	0.38 ± 0.02	0.17 ± 0.01
	WKS + PCA100	0.77 ± 0.1	0.44 ± 0.06	2.21 ± 0.23	0.36 ± 0.01	0.14 ± 0.01
HVA	Con	222.91 ± 16.55	81.12 ± 2.4	196.17 ± 7.1	**80.36 ± 2.19**	**86.91 ± 2.92**
	WKS	196.76 ± 7.1	76.02 ± 1.99	198.31 ± 7.62	72.97 ± 2.06 *	72.78 ± 2.88 ***
	WKS + PCA50	183.87 ± 7.43	98.63 ± 13.03	181.56 ± 7.37	70.48 ± 2.36 *	70.46 ± 3.13 ***
	WKS + PCA100	173.24 ± 20.32	83.39 ± 3.02	178.62 ± 8.24	71.05 ± 3.51*	73.24 ± 2.88 ***
HVA/	Con	2.07 ± 0.46	2.99 ± 0.4	11.22 ± 1.4	**1.48 ± 0.04**	1.90 ± 0.09
DA	WKS	1.77 ± 0.46	2.44 ± 0.31	16.38 ± 3.61	1.29 ± 0.06 *	1.78 ± 0.07
	WKS + PCA50	1.77 ± 0.37	2.18 ± 0.37	13.97 ± 2.53	1.28 ± 0.03 *	1.69 ± 0.04
	WKS + PCA100	2.43 ± 0.4	2.97 ± 0.52	16.6 ± 2.44	1.30 ± 0.06 *	1.76 ± 0.07
NA	Con	1271.21 ± 231.25	552.44 ± 28.45	433.32 ± 12.78	572.54 ± 11.01	292.85 ± 6.07
	WKS	1353.06 ± 184.68	509.64 ± 30.33	402.78 ± 20.17	595.31 ± 12.15	299.63 ± 15.28
	WKS + PCA50	1116.44 ± 159.15	552.42 ± 36.8	373.00 ± 13.93	586.34 ± 10.01	296.35 ± 8.71
	WKS + PCA100	865.37 ± 187.56	537.88 ± 49.22	395.95 ± 13.66	569.62 ± 14.17	280.16 ± 6.53
MHPG	Con	3.84 ± 0.62	2.32 ± 0.26	**1.67 ± 0.31**	**7.19 ± 0.32**	2.60 ± 0.25
	WKS	3.08 ± 0.57	1.76 ± 0.14	3.50 ± 0.61 ***	6.03 ± 0.36 **	2.68 ± 0.25
	WKS + PCA50	4.02 ± 0.66	1.56 ± 0.22	5.15 ± 0.29 ****•	5.96 ± 0.22 *	2.28 ± 0.22
	WKS + PCA100	1.91 ± 0.42	2.00 ± 0.35	3.82 ± 0.34 ***^	5.86 ± 0.19*	1.99 ± 0.21

* *p* < 0.05 vs. Con, ** *p* < 0.01 vs. Con *** *p* < 0.005 vs. Con, **** *p* < 0.001 vs. Con, **•** *p* < 0.05 vs. WKS, ^ *p* < 0.05 vs. WKS + PCA50.

**Table 4:** Effect of long-term oral administration of PCA at doses of 50 mg/kg and 100 mg/kg on amino acid levels (mean ± SEM) (ng/mg tissue) in PTD model of WKS in rats.

Amino Acid	Group	Hypothalamus	Hippocampus	Prefrontal Cortex	Medulla Oblongata	Spinal Cord
TAU	Con	487.13 ± 24.6	858.97 ± 15.67	1067.58 ± 36.00	231.51 ± 2.23	183.57 ± 8.1
	WKS	479.81 ± 23.34	851.18 ± 19.63	1100.47 ± 34.5	225.28 ± 2.12	167.17 ± 5.96
	WKS + PCA50	457.31 ± 27.9	879.43 ± 26.56	1127.26 ± 38.44	237.53 ± 3.17	161.42 ± 5.45
	WKS + PCA100	437.5 ± 48.5	842.73 ± 23.02	1043.9 ± 26.86	230.78 ± 3.97	161.07 ± 5.45
HIS	Con	19.64 ± 4.03	13.22 ± 1.1	11.88 ± 0.31	5.04 ± 0.17	7.43 ± 0.5
	WKS	12.23 ± 0.66	12.59 ± 0.43	12.48 ± 0.92	5.00 ± 0.20	11.39 ± 5.31
	WKS + PCA50	38.42 ± 25.63	17.42 ± 2.15	13.24 ± 0.66	5.43 ± 0.11	7.68 ± 1.32
	WKS + PCA100	13.64 ± 2.61	14.15 ± 0.8	12.78 ± 1.06	5.19 ± 0.13	7.12 ± 0.54
SER	Con	88.35 ± 4.34	123.09 ± 5.22	136.59 ± 4.44	48.82 ± 0.70	**65.84 ± 3.18**
	WKS	83.53 ± 4.26	119.68 ± 5.02	143.59 ± 4.22	47.89 ± 0.87	57.66 ± 1.98 *
	WKS + PCA50	80.72 ± 4.04	129.54 ± 6.36	145.53 ± 4.31	49.08 ± 1.07	55.02 ± 1.92 ***
	WKS + PCA100	73.79 ± 7.79	127.63 ± 4.48	140.97 ± 3.20	48.05 ± 0.81	53.92 ± 1.56 ***
ASP	Con	414.65 ± 16.28	283.09 ± 16.2	455.5 ± 17.45	412.75 ± 5.19	359.24 ± 7.57
	WKS	378.88 ± 15.73	242.02 ± 14.5	474.05 ± 16.04	391.57 ± 6.57	340.95 ± 12.66
	WKS + PCA50	392.77 ± 15.25	262.51 ± 12.98	438.55 ± 14.77	396.67 ± 6.28	357.07 ± 7.44
	WKS + PCA100	356.05 ± 37.97	272.01 ± 19.09	476.04 ± 7.69	409.31 ± 7.52	364.95 ± 10.45
ALA	Con	74.49 ± 3.39	99.01 ± 2.28	n/d	**47.51 ± 1.62**	**47.06 ± 2.27**
	WKS	66.63 ± 2.52	91.69 ± 1.55		42.79 ± 1.04 **	40.99 ± 1.55
	WKS + PCA50	68.82 ± 3.12	97.83 ± 3.52		**50.2 ± 1.14 ••••**	38.72 ± 1.59 *
	WKS + PCA100	64.85 ± 7.00	95.19 ± 3.23		**49.58 ± 0.96 •••**	42.57 ± 2.21
GLU	Con	n/d	1601.9 ± 45.64	1985.52 ± 60.30	**864.12 ± 6.49**	756.23 ± 20.51
	WKS		1491.4 ± 43.13	1893.71 ± 54.31	819.8 ± 11.51 **	700.31 ± 20.3
	WKS + PCA50		1571.26 ± 51.97	1856.07 ± 70.24	**858.36 ± 8.87 ••**	699.9 ± 15.59
	WKS + PCA100		1551.25 ± 41.31	1875.73 ± 65.19	**880.98 ± 9.89 ••••**	726.5 ± 14.67
GABA	Con	685.22 ± 73.43	265.56 ± 10.67	304.09 ± 8.75	187.72 ± 3.17	116.91 ± 5.58
	WKS	623.73 ± 71.04	253.99 ± 9.28	371.77 ± 45.38	178.84 ± 3.64	108.08 ± 3.98
	WKS + PCA50	693.21 ± 64.03	293.62 ± 20.24	343.6 ± 12.59	189.95 ± 3.78	104.71 ± 4.38
	WKS + PCA100	523.66 ± 76.69	252.95 ± 12.52	318.41 ± 6.83	189.12 ± 4.02	108.31 ± 5.75
THR	Con	112.39 ± 3.82	**96.98 ± 4.37**	96.40 ± 2.15	**122.83 ± 2.72**	**114.6 ± 2.97**
	WKS	104.35 ± 4.96	84.52 ± 1.36 *	94.86 ± 2.51	114.23 ± 2.31 *	101.96 ± 2.71 *
	WKS + PCA50	111.91 ± 2.98	92.53 ± 1.97	97.96 ± 2.42	114.87 ± 1.91 *	106.03 ± 3.06 *
	WKS + PCA100	93.57 ± 11.23	85.23 ± 2.33 *	94.02 ± 2.28	108.74 ± 2.08 ****	103.32 ± 2.76 *

* *p* < 0.05 vs. Con, ** *p* < 0.01 vs. Con *** *p* < 0.005 vs. Con, **** *p* < 0.001 vs. Con, **••** *p* < 0.01 vs. WKS, **•••** *p* < 0.005 vs. WKS, **••••** *p* < 0.001 vs. WKS, n/d—no data (below detection threshold).

## Data Availability

The data are available from the corresponding author upon reasonable request.

## Abbreviations

5-HIAA	5-hydroxyindoleacetic acid
5-HT	5-hydroxytryptamine (serotonin)
AGEs	Advanced glycation end products
ALA	Alanine
ANOVA	Analysis of variance
ASP	Aspartic acid
b.w.	Body weight
Con	Healthy control group
CNS	Central nervous system
DA	Dopamine
DOPAC	3,4-dihydroxyphenylacetic acid
EAAT1, GLAST	Astrocytic glutamate transporters 1
EAAT2, GLT-1	Astrocytic glutamate transporter 2
FF	Foot Fault test
GABA	γ-aminobutyric acid
GH	Post-hoc Games–Howell test
GLU	Glutamate
HB	Hole–Board test
HIS	Histidine
HPLC-ED	High-performance liquid chromatography with electrochemical detection
HVA	Homovanillic acid
LRR	Loss of righting reflex
MHPG	3-methoxy-4-hydroxyphenylglycol
MWM	Morris Water Maze test
NA	Noradrenaline
NK	Post-hoc Newman–Keuls test
NOR	Novel Object Recognition test
OF	Open Field test
PCA	Protocatechuic acid
PCA50	PCA administered at dose 50 mg/kg b.w.
PCA100	PCA administered at dose 100 mg/kg b.w.
PTD	Pyrithiamine-induced thiamine deficiency
SER	Serine
TAU	Taurine
TD	Thiamine deficiency
THR	Threonine
WKS	Wernicke–Korsakoff syndrome
